# Sensor Fusion for the Robust Detection of Facial Regions of Neonates Using Neural Networks

**DOI:** 10.3390/s23104910

**Published:** 2023-05-19

**Authors:** Johanna Gleichauf, Lukas Hennemann, Fabian B. Fahlbusch, Oliver Hofmann, Christine Niebler, Alexander Koelpin

**Affiliations:** 1 Nuremberg Institute of Technology, 90489 Nuremberg, Germanychristine.niebler@th-nuernberg.de (C.N.); 2 Division of Neonatology and Pediatric Intensive Care, Department of Pediatrics and Adolescent Medicine, Friedrich-Alexander-Universität Erlangen-Nürnberg, 91054 Erlangen, Germany; fabian.fahlbusch@uk-erlangen.de; 3 University Children’s Hospital Augsburg, Neonatal and Pediatric Intensive Care Unit, 86156 Augsburg, Germany; 4 Hamburg University of Technology, 21073 Hamburg, Germany; alexander.koelpin@tuhh.de

**Keywords:** non-contact monitoring, neonates, sensor fusion, neural network, face detection

## Abstract

The monitoring of vital signs and increasing patient comfort are cornerstones of modern neonatal intensive care. Commonly used monitoring methods are based on skin contact which can cause irritations and discomfort in preterm neonates. Therefore, non-contact approaches are the subject of current research aiming to resolve this dichotomy. Robust neonatal face detection is essential for the reliable detection of heart rate, respiratory rate and body temperature. While solutions for adult face detection are established, the unique neonatal proportions require a tailored approach. Additionally, sufficient open-source data of neonates on the NICU is lacking. We set out to train neural networks with the thermal-RGB-fusion data of neonates. We propose a novel indirect fusion approach including the sensor fusion of a thermal and RGB camera based on a 3D time-of-flight (ToF) camera. Unlike other approaches, this method is tailored for close distances encountered in neonatal incubators. Two neural networks were used with the fusion data and compared to RGB and thermal networks. For the class “head” we reached average precision values of 0.9958 (RetinaNet) and 0.9455 (YOLOv3) for the fusion data. Compared with the literature, similar precision was achieved, but we are the first to train a neural network with fusion data of neonates. The advantage of this approach is in calculating the detection area directly from the fusion image for the RGB and thermal modality. This increases data efficiency by 66%. Our results will facilitate the future development of non-contact monitoring to further improve the standard of care for preterm neonates.

## 1. Introduction

Neonates on the Neonatal Intensive Care Unit (NICU), especially those born preterm, require continuous monitoring (e.g., via electrocardiogram (ECG), pulse oximeter or temperature probes) of their vital signs (heart rate, respiratory rate and body temperature). Most of these techniques, however, rely on direct skin contact which might pose a clinical problem particularly for immature neonates with commonly increased skin sensitivity. Clinically, this can lead to pressure marks, eczema, skin irritations and even epidermal abrasion [1]. Hence, current research aims to develop non-contact monitoring approaches to improve neonatal comfort. Technically, monitoring of vital signs requires a reliable detection of the head and facial regions such as the nose. Unfortunately, the most common face detection methods are only utilized on adults and cannot be readily applied to the body proportions of neonates [2]. Moreover, sufficient open-source data of neonates on the NICU is lacking. In the clinical setting, non-contact monitoring is challenged by incubator-related issues such as the varying quality of lighting and the positioning of detectors in close proximity to the area of interest. Therefore, our aim is to collect thermal and Red Green Blue (RGB) camera data of incubator-nursed (preterm) neonates on the NICU. Thermal cameras measure temperature gradients and display it within a false color image and are light independent. RGB cameras collect visible light and display it how a human would perceive it. RGB camera images hold more features than thermal camera images. The idea is to fuse the different image modalities to allow a robust face detection under all conditions. A neural network shall be trained with the fused data.

### 1.1. State of the Art

In this chapter, we describe the State of the Art for thermal-RGB-fusion approaches as well as neural networks for face detection.

#### 1.1.1. Thermal-RGB-Fusion

Direct and indirect approaches for the fusion of an RGB with a thermal camera will be presented in the following section.

##### Direct Extrinsic Calibration

St. Laurent et al. use the angles and the ratio of the field of view (FoV) of the thermal and the RGB camera [3]. The calibration is facilitated using parallel or convergent optical axes of the cameras. Shivakumar et al. use a projection of the RGB coordinates into the 3D space using a depth image which is determined by stereo depth calculations [4]. Their RGB camera is a Stereolabs Zed Mini (Stereolabs, San Francisco, CA, USA). The 3D coordinate is then projected into the thermal camera image. For the intrinsic calibration, a checkered pattern is used. Yang et al. first detect the centers of the circles of the calibration target within the RGB and the thermal camera image [5]. The root mean square error (RMSE) of the coordinates of the detected circle centers is calculated. Finally, normalized cross correlation (NCC) is used as an image registration procedure.

##### Indirect Extrinsic Calibration

Since it is difficult to achieve a high precision using a direct extrinsic calibration, approaches for an improved extrinsic calibration using a third sensor will be discussed. Krishnan and Saripalli apply a cross calibration of an RGB camera with a thermal camera with a light detection and ranging (LIDAR) sensor [6]. First, the RGB camera is calibrated with the LIDAR. The transformations between the edges of the checkered calibration target within the camera image and the 3D point cloud are determined. The extrinsic calibration between the thermal camera and the LIDAR is determined in the same manner using a different calibration target which is visible within the thermal camera image. Gleichauf et al. employed a thermal-RGB-laser fusion for a project with the German railway [7]. In this case, no direct fusion between the RGB and thermal camera takes place, but the single systems are fused with the 3D laser scanner by themselves. The points of the laser scanner are projected into the two image modalities.

##### Thermal-ToF-Fusion

Tisha et al. fuse a thermal camera with a Kinect2. First, the intrinsic calibration takes place and in the next step the homography of both cameras is calculated (intrinsic times extrinsic). The multiplication of the homographies with a pixel point of the camera delivers the pixel point within the other camera [8]. Van Baar et al.’s extrinsic calibration approach is based on the publication by Yang et al. [9]. They fuse an RGB image with a depth image. For the thermal camera a checkered pattern with heated resistors is used [10]. Cao et al. fuse a thermal with a Red, Green, Blue -Depth (RGB-D) camera (Kinect2) using the thermal-guided iterative closest point (T-ICP) algorithm [11]. A thermal-3D-pointcloud registration is applied. The resulting error is defined by a nonlinear least-squares objective and minimized by the Gauss–Newton method. Pfitzner fuses a thermal camera with a Kinect via the infrared sensor frames of the Kinect to the thermal camera and the Kinect2. For the fusion the RGB images of the Kinect camera systems are used [12]. The method by Rocco Spremolla is very similar, where first the intrinsic calibrations of the thermal and the Kinect2 cameras are determined and then the relative poses of thermal/RGB (Kinect) to the infrared (IR)/Depth of the Kinect. The transformations can then be calculated [13].

##### RGB-ToF-Fusion

Similarly fusion approaches between ToF and RGB cameras exist. Salinas et al. recommend using a depth dependent homography lookup table instead of calculating the extrinsic parameters of the ToF and RGB camera. In both modalities, point correspondences need to be found [14]. Kim et al. use five RGB and three ToF cameras which are set up in a semicircle. The intrinsic and extrinsic calibration can be calculated using the intensity image of the ToF camera but inaccurate registrations of the depth images can occur [15]. The aim is to achieve a precise 3D reconstruction of surfaces.

##### Fusion Using Neural Networks

There are several approaches using neural networks for generating fusion images. In each case, the images are taken from a long distance [16]. The network used by Alexander et al. is used for civil infrastructure applications and states that the robustness increases for fusion images [17]. Jung et al. use a neural network for the creation of fusion images out of near infrared (NIR) and noisy RGB images [18]. Wang et al. apply a multi-channel convolutional neural network (CNN) for infrared and visible image fusion using images of the same scene and position [19]. Another approach uses salience detection and a CNN for fusion image generation [20]. Using neural networks for the sensor fusion requires a lot of data to train the networks. As we demonstrated in this article, all of these fusion approaches are only applicable for long distance environments.

#### 1.1.2. Face Detection Using Neural Networks and Image Processing

For adults, there are a lot of scientific contributions which address the detection of body regions. The field of application can differ and is not restricted to the monitoring of vital signs. The detection of the head and face became more popular after the publication of the WiderFace dataset [21]. Face detection developed rapidly and mainly focused on extreme and real variation problems including scaling, pose, occlusion, lighting conditions, blur, etc. Neural networks are mainly used for the common object detection and trained for the special case of face detection with the WiderFace dataset and adjusted. Known methods are YOLO5Face [22], RetinaFace [23], MTCNN [24], Mask R-CNN [25] and an implementation of the Faster R-CNN [26]. These are all adaptions of the original architecture (trained on RGB data of the WiderFace dataset) especially designed to overcome the problems of the face detection. Some were extended such that the pose or the orientation of the face can be detected. The dataset also holds images of babies but they are neither newborns nor recorded within the clinical field. Most of the dataset consists of images of adults. Therefore, nets which were trained on the WiderFace dataset are not suitable for detecting the face region of neonates within the incubator. There are many other models which were trained with the WiderFace dataset. An overview can be found on the WiderFace website [21].

There is little research on the head detection within thermal images. Cheong et al. [27] use the Otsu threshold method to convert thermal into binary images. Using the horizontal projection of the images the global minimum can be determined which helps to identify the height and width of the head region.

Kopaczka et al. [28] use a so-called Histogram of oriented gradients detector for the evaluation of thermal images. They also compare other machine learning methods and classic approaches within image processing. They conclude that machine learning methods deliver better results than traditional approaches.

Silva et al. [29] use deep learning methods such as the neural network YOLOv3 by Redmon et al. [30]. They apply transfer learning in order to implement a network trained on RGB data to detect heads within thermal images.

A similar approach can be found with Vuković et al. [31]. They use a R-CNN which delivers very good results but has no real-time capability.

Mucha et al. [32] implement the SCRFD DL architecture by Guo et al. [33] which returned satisfactory precision values for the WiderFace Challenge. SCRFD DL was adapted such that faces can be detected within thermal images.

#### 1.1.3. Face Detection for Neonates

As the proportions of adult faces differ from the faces of neonates the previously presented methods cannot be used for neonates without further adaptions [2]. Chaichulee et al. use a self designed multi-task CNN which segments the visible skin of the neonate (clinical field) within the RGB image [34]. In most cases the torso and the head are made visible. These regions are used to determine the respiratory and heart rate.

Green et al. [35] extended the network such that the head, the torso and the nappy area can be detected. It is based on the Faster R-CNN [36]. The whole network is made up of a core network based on VGG16 [37], three branches for the patient detection and skin segmentation with movement control and a branch for the detection of the body regions. The average precision achieved for the “head” class is 98.2. The data set holds 2269 images of 15 subjects of different ethnic backgrounds and sex. The recordings were taken within an incubator during the stay on the NICU.

Kyrollos et al. use the RetinaNet [38], to detect the thorax and face region of neonates in an RGB video stream [39]. A model was trained with a transfer learning approach by applying weights which were pre-trained on the ImageNet dataset [40]. Three different core nets were tested: ResNet50, ResNet101 and ResNet152. There were only small differences between the three models so it was decided to use the fastest model, ResNet50. A mean average precision of 56.9 was achieved. The results of the detection are used for the calculation of the respiratory rate. The RGB images (200 images per subject with 5 subjects) were not taken within an incubator.

A CNN with pyramidal hierarchic features is presented by Lu et al. [41]. First, an adaption of VGG16 [37] is used to extract the implicit features of the normalized image. Multi-scale feature maps are selected to predict and detect different sizes of facial features of neonates. The third part of the system contains two parallel branches: a classification branch of facial and non-facial regions and a regression branch for the position of the face. The dataset holds 3600 images with different perspectives, gestures, facial expressions and backgrounds. If the images were taken on a NICU is unknown. An average precision of 92.7 for the facial region was achieved.

An often-used model for the detection of neonates is YOLOv3 [30] and YOLOv5 [42]. In Nagy et al.’s work, YOLOv3 is the basis for the detection of head, torso and hands of a nurse or parents [43]. In combination with a LSTM (Long Short-Term Memory) the static objects within the RGB video can be detected. The pulse and respiratory rate are determined from the results of the classification block (YOLOv3 plus LSTM). The author’s dataset holds 96 h of labeled RGB video data recorded on a NICU. The achieved precision of the object detection is not stated in the publication. The results of the classification block made up of YOLOv3 and LSTM show a sensitivity of 97.9, specificity of 97.5 and an F1 score of 98.1. Therefore, the object detection should also deliver high levels of precision.

Khanam et al. [44] use Redmon’s YOLOv3 for the face detection of preterm neonates in the incubator. The head needs to be detected during normal lighting conditions as well as during phototherapy with ultraviolet (UV) light. The dataset holds 473 images from the internet. A transfer learning approach was chosen. The starting point were the weights of a YOLOv3 net trained on the MS COCO dataset. The authors do not state the precision of the face detection.

By training YOLOv5 on a proprietary RGB dataset with labeled faces of newborns in the clinical field Hausmann et al. almost reach real-time face detection [2]. The results show a precision of 68.7. For the dataset, the University of South Florida Multimodal Neonatal Pain Assessment Dataset (USF-MNPAD-I) [45] was used. In comparison, a net was also trained with the WiderFace dataset, which led to a precision of 7.37.

Dosso et al. [46] also use YOLOv5. They compare different face detection models such as RetinaFace [23] and YOLO5Face [22] which were trained with the WiderFace dataset. By using transfer learning approaches with their own dataset they reach a precision of 81.45. The best results were achieved by the fine-tuned YOLO5Face which they called NICUFace. The dataset contained 33 subjects which were filmed over 153 h. They also use a thermal camera but its data were not used for their work.

Antink et al. [47] follow another approach by extending an encoder decoder method through a modified version of the ResNet50 encoder. Their aim is to segment different regions of the body of preterm neonates. They use a freely available dataset holding segmentation data of adults. A second dataset containing NIR and RGB images of preterms on the NICU was used for transfer learning. The head is well segmented within the RGB images (Intersection over Union (IoU) of 82.0). For the torso, an IoU of 41.0 is reached. Within the NIR images the detection delivered less accurate results. By applying methods of data augmentation and generating artificial NIR images an IoU of 62.0 for the head detection and of 14.0 for the torso was achieved. Voss et al. extended this research by using a U-Net architecture for training with RGB, NIR and fusion images [48]. Their fusion does not take place on the images itself, but on the feature level. They reach a mean IoU of 85.0 with their fusion model. The results for the RGB model is similar with 85.0. Within the NIR images the accuracy drops significantly to a mean IoU of 75.0. In all modalities, the segmentation of the torso performed worst.

Beppu et al. research the detection of different body regions within thermal images [49]. They trained YOLOv5 [42] to detect six body regions such as the head, torso, arms and legs. For the dataset, 4820 thermal images of 26 different subjects extracted out of 1872 h of video material were used. The images were taken within an incubator on a NICU. The head detection reached a precision of 94.8, whereas the torso an average precision (AP) of 95.8. They use the detected regions to determine the body temperature of preterms.

Besides the deep learning methods there are other approaches for the face detection. Awais et al. [50] solves the problem of the face detection using the CIE L*a*b intensity based detection by Neophytou et al. [51] and Fairchild [52] within their framework NFAR (Neonatal Face Attributes Recognition) for RGB images. NFAR reaches a precision of up to 94.9.

No literature regarding the face detection of neonates with fused images currently exists.

#### 1.1.4. Face Detection Using Fused Images

Two sensor fusion approaches for the face detection based on thermal and visual image data are introduced by Bebis et al. [53]. The first approach is pixel based which is applied within the wavelet plane, the other one is feature based in the Eigenspace. For the wavelet method, differences within the resolution are detected, for the Eigenspace global image features are fused. The Eigenspace method is based on a Principal Component Analysis (PCA). The aim is to calculate a fused image out of the infrared and the RGB data and to use the most significant features in both spectra. The heat energy which is emitted by the face can be measured by the thermal camera. The reflection of light from the facial surface is detected by the visual camera. A simple pixel to pixel fusion has the disadvantage that spacial information is lost. If the fusion takes place for different resolution planes, features with different spacial dimensions can be used, especially the most significant ones. Both approaches were tested by Bebis et al. with the Equinox dataset [54] with long wave infrared (LWIR) and RGB camera images. The dataset only holds head-on images of adults faces. In our case, it has to be trained with facial data of neonates. In comparison, the fusion within the wavelet space delivers better results, but the computational expense is higher. In the future, the authors want to use a hair-wavelet-transformation [53]. Both of these methods have only been tested on adults and do not necessarily use the fused images for the face detection or the vital sign detection.

Chen et al. propose a neural network for creating fusion images for face recognition [55]. They state that the recognition improves when fusing data. The network is only trained on adults.

#### 1.1.5. Neural Networks Using Fused Image Data

There are a few approaches using neural networks with fused image data as input. The applications lie within the perception for automated driving [56] and pedestrian detection (Faster R-CNN) [57]. In both cases, the fusion images are also created by a neural network. Shopovska et al. propose that the fusion generates more informative images [57].

There are so-called RGB-T trackers which track objects in the foreground using neural networks within RGB thermal images. Thus, in contrast to image fusion, the fusion precision is less important [58,59].

There are no neural networks using fused image data for the face detection for neonates. We will now present a method addressing this gap.

## 2. Material and Methods

We now describe all parts which are required for our sensor fusion as well as the training of the neural networks for neonatal face detection. First, the theoretical approach is presented, and then the hardware and software are described in more detail. The measurement series we recorded will be presented in the last step.

### 2.1. Concept and Theoretical Approach

In this section, the concept of our fusion approach, the architecture of our neural networks and the theory behind the sensors used is presented.

#### 2.1.1. Sensors

First, the theory behind the thermal, RGB and the 3D-ToF camera are described. A detailed description of the hardware used will be presented in the following section.

Thermal cameras are 2D infrared thermometers which detect the emitted heat of objects. Within the thermal camera image temperature gradients are displayed as false color images. RGB cameras collect visible light and display the image how a human being would perceive it [60].

Within time-of-flight cameras two principles can apply: the phase difference method and the impulse time-of-flight method. In our ToF camera the phase difference method is used [61]. This is based on the phase shift caused by the reflected modulated signal. Using the phase shift ϕ and the wavelength λ of the modulated signal the distance to the object can be calculated:

(1) $$\begin{array}{c} d=\frac{\phi }{2\;\cdot \;\pi }\;\cdot \;\frac{\lambda }{2} \end{array}$$

#### 2.1.2. Intrinsic Calibration

Fusion between the different sensors requires prior intrinsic calibration of the thermal and RGB camera. During the rectification the tangential and radial distortion coefficients are calculated and removed. It relates to the projection of the chip plane onto the image plane of the camera sensor such that the transformation between the sensor’s pixel coordinates and the world coordinate frame is given. Both cameras can be modelled as a pinhole camera model represented by this formula:

(2) $$\left(\begin{array}{c} u\\ v\\ 1 \end{array}\right)=\left(\begin{array}{ccc} f_{x} & 0 & u_{0}\\ 0 & f_{y} & v_{0}\\ 0 & 0 & 1 \end{array}\right)\left(\begin{array}{c} X/Z\\ Y/Z\\ 1 \end{array}\right)$$

with $f_{x}$ and $f_{y}$ as focal length, $u_{0}$ and $v_{0}$ as center of the camera sensors. *u* and *v* are the *x*- and *y*-coordinate within the image. *Z* is a scaling factor.

Images of the calibration target have to be processed from different positions with different distances and skew so that the distortion coefficients can be calculated as precisely as possible. The rectification can be calculated using the following formula:

(3) $$\begin{array}{c} \left(\begin{array}{c} u\;\cdot \;\left(1+k_{1}r^{2}+k_{2}r^{4}+k_{3}r^{6}\right)+2p_{1}v+p_{2}\;\cdot \;\left(r^{2}+2u^{2}\right)\\ v\;\cdot \;\left(1+k_{1}r^{2}+k_{2}r^{4}+k_{3}r^{6}\right)+2p_{1}\;\cdot \;\left(r^{2}+2v^{2}\right)+p_{2}u \end{array}\right) \end{array}$$

with $k_{1}$, $k_{2}$ and $k_{3}$ as radial distortion coefficients and $p_{1}$ and $p_{2}$ as tangential distortion coefficients. *r* is the distance between *u* and *v*.

#### 2.1.3. Sensor Fusion

In the following, the concept of our sensor fusion approach will be described. The precision of direct fusion between the thermal and RGB camera is limited to its specific calibration distance [3]. This is not ideal due to the varying sizes of the neonates the distance to the cameras can differ. Therefore, it is our aim to develop an indirect fusion approach using the Time of Flight camera of our sensor setup from [62] as the third sensor. The advantage of the 3D ToF camera is that it can take the depth into account. The following steps are necessary for the indirect thermal-RGB-fusion:

- Detect circles of the calibration target within the ToF mono image and calculate the corresponding depth points.
- Detect circles within RGB and thermal image.
- Calculate transformation between RGB and ToF camera using the circle centers.
- Calculate transformation between thermal and ToF camera using the circle centers.
- Project RGB points into the thermal image (at the position of the ToF points).

#### 2.1.4. Neural Networks RetinaNet and YOLOv3

Now the architecture of the neural networks RetinaNet and YOLOv3 will be presented.

##### RetinaNet

RetinaNet is a CNN developed by Facebook AI Research (FAIR) and published by Lin et al. in [38]. It is known as one of the best one-stage object detection models that is proven to work well with dense and small scale objects.

**Figure 1:**
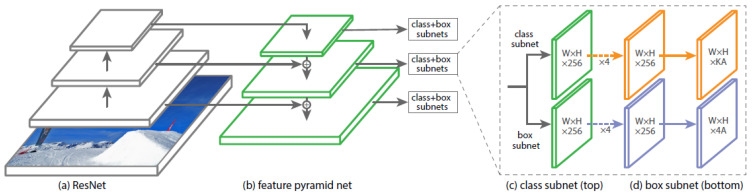
Structure of RetinaNet from the original paper [38]. Reprinted with permission from [38]. 2018, Lin et al.

RetinaNet is composed of several networks, as displayed in Figure 1. On top of a Residual Network (ResNet) (a) [63] backbone a Feature Pyramid Network (FPN) (b) [64] is used. ResNet utilizes skip connections to extract deep features [63]. An FPN is used on top of ResNet for constructing a rich multiscale feature pyramid from one single resolution input image [64]. Lateral connections allow RetinaNet to merge feature maps with the same resolution. The feature pyramid is included to improve the accuracy of detection for objects of a different scale. In the original paper [38], five different scales are used. On top of the FPN, two subnets are added: first, a classification subnet (c) which predicts the probability of the presence of an object at each position for each anchor and object class. The second subnet is the bounding box regression subnet (d) with the purpose of regressing the offset of an anchor box to a nearby ground truth object.

It has been shown that there is an extreme imbalance between the foreground and background classes in a single-stage detector [38]. To overcome this problem, RetinaNet uses a special loss function, called Focal Loss. It is used on the output of the classification subnet. By using Focal Loss, less loss is produced by “easy” negative samples (such as the background), so that the loss is focused on “hard” samples (real objects we want to detect), which improves prediction accuracy. Focal Loss reduces the cost of simple detections and increases the cost of correcting misclassified objects.

RetinaNet achieves state-of-the-art performance and outperforms most one-stage and two-stage detectors, such as Fast Region-Based Convolutional Neural Networks (R-CNN) [65] or Faster R-CNN [36].

##### YOLOv3

“You only look once”, or YOLO, is one of the faster object detection algorithms found in the literature. YOLOv3 is the third revised version of YOLO published by Redmon et al. in 2018 [30].

**Figure 2:**
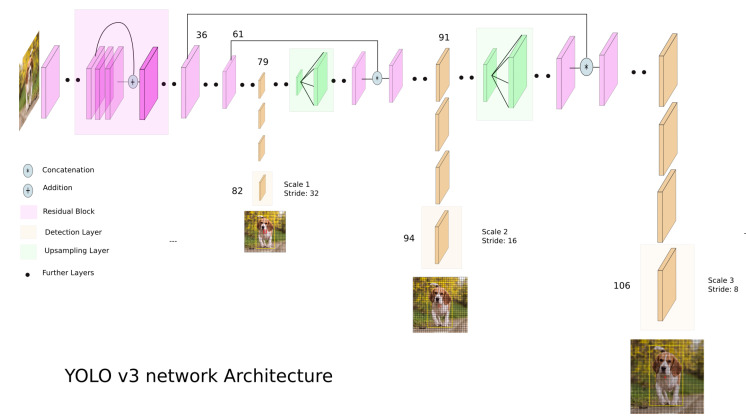
YOLOv3 structure from Ayoosh Kathurias blog post in Towards Data Science [67]. Reprinted with permission from [67]. 2018, Ayoosh Kathuria.

The structure of YOLOv3 relies on similar elements as RetinaNet. The backbone is Darknet-53, a fully convolutional network with 53 layers. Like ResNet, it uses so-called skip connections to bypass individual layers during training in order to enable a deeper architecture. Darknet is used for feature extraction. Compared with that of ResNet, the network structure used by Darknet-53 better utilizes the graphical processing unit (GPU), making it more efficient to evaluate and thus faster. For the task of detection, 53 additional layers are added on top of Darknet, so that YOLOv3 is based on a fully convolutional architecture with 106 layers. The overall structure is shown in Figure 2.

A major innovation of YOLOv3 in contrast to its predecessors [66] is that predictions are performed at three different scales. The 82nd layer performs the first detection. During the passage of the previous 81 layers, the input image is down-sampled by a factor of 32.

After a few convolutional layers, the 79th layer is deeply concatenated with the 61st layer sampled upward by two. The resulting layer is then subjected to further convolutional layers until another detection is performed in layer 94. The prediction is thus performed on a feature map that is smaller than the initial image by a factor of 16.

The last detection is then performed in layer 106, which corresponds to a down-sampling factor of 8. Similarly to the previous prediction head, a deep convolution is applied. The 91st layer is up-sampled by a factor of two and then used to convolute layer 36.

In their paper, Redmon et al. tried to use Focal Loss as a loss function. However, this did not bring any further improvements. YOLOv3, on the other hand, relies on logistic regression to determine the object confidence and class predictions [30].

YOLOv3 is currently considered to be one of the fastest object detection algorithms and is a good choice if real-time detection is needed, with negligible loss of accuracy.

#### 2.1.5. Data Augmentation

In general, data augmentation methods can be divided into two categories: traditional and advanced methods. While advanced methods such as Generative Adversial Networks (GANs) are computationally intensive, traditional methods have a low complexity time [68]. In this paper, we focus on the use of different traditional data augmentation techniques due to their ease of use. All data sets are subjected to data augmentation. The parameters were empirically adjusted to the specific properties of the image types.

##### Mirroring

**Figure 3:**
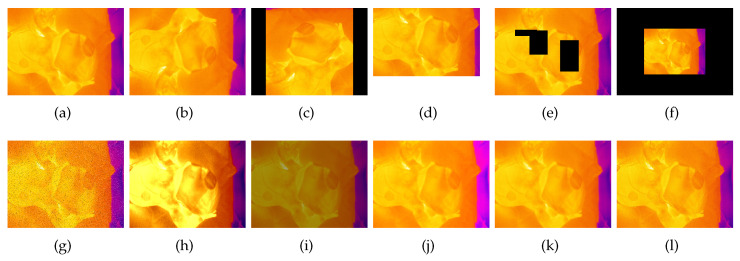
Augmented thermal images: (**a**) original; (**b**) vertical mirroring; (**c**) rotation; (**d**) random crop; (**e**) erasing; (**f**) zooming; (**g**) salt/pepper; (**h**) histogram equalization; (**i**) contrast; (**j**) saturation; (**k**) blurring; (**l**) sharpening [69].

**Figure 4:**
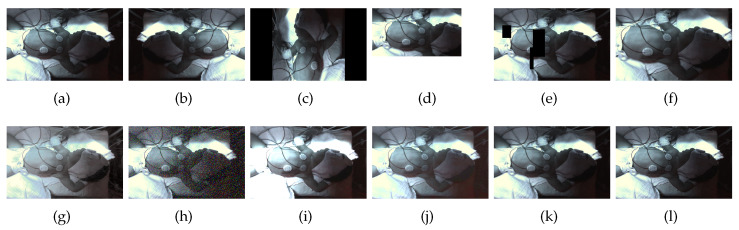
Augmented RGB images: (**a**) original; (**b**) horizontal mirroring; (**c**) rotation; (**d**) random crop; (**e**) erasing; (**f**) zooming; (**g**) histogram equalization; (**h**) Gaussian; (**i**) contrast; (**j**) saturation; (**k**) blurring; (**l**) sharpening [69].

The mirroring is performed by swapping the individual pixel values on the basis of the symmetry axes. Either a single mirroring on x- or y-axis or a double mirroring on both is accomplished. In addition to the single images, the bounding box is also transformed to prevent relabeling. Obtained images are shown in Figure 3b and Figure 4b.

##### Rotation

The rotation operation uses degree increments of 90${}^{\circ }$ between 0${}^{\circ }$ and 360${}^{\circ }$. Other angles were neglected here, as it would have entailed relabeling. To maintain the proportions of the image content, the rotated images are then resized back to their original size. For the thermal and fused images, this corresponds to a size of 640 × 480, for the RGB images the total size is 1146 × 716. This can be seen in Figure 3c and Figure 4c.

##### Zooming

This transformation is dedicated to zooming in or out to the center of an image. It returns an enlarged or shrunk view of the image without changing the image dimensions. The zoom factor is set at random between the values 0.5 and 1.5. This corresponds to a reduction or enlargement of 50%. Zooming is also applied on the bounding boxes to avoid relabeling. Images transformed with zooming can be seen in Figure 3f and Figure 4f.

##### Random Crop

Random crop is similar to zooming. The difference is that instead of zooming in or out of the center of the image, a randomly generated section of the image is selected. This is then used as the new image. The bounding box is either recalculated or discarded if less than 75% of the area is in the image section. The image dimensions are retained. The image section is moved to the upper left corner of the new image. The rest of the image is filled in with black (see Figure 3d and Figure 4d).

##### Erasing

This transformation randomly erases rectangular areas of different sizes within an image. If bounding boxes with an overlap of more than 50% are deleted, they are discarded. Up to three of these rectangles are applied per image. Sample images can be viewed in Figure 3e and Figure 4e.

##### Noise

Several genres of noise can be utilized for data augmentation. In this case, either salt-and-pepper (Figure 3g) or Gaussian noise (Figure 4h) is added to the original image. Salt-and-pepper noise is known as impulse noise. It presents itself as sparsely occurring white and black pixels. The transformation is irrelevant for the bounding boxes, so they do not need to be changed.

##### Contrast and Saturation Changing

The contrast of individual images is changed by multiplying all pixel values by a random constant between 0.5 and 0.75 (see Figure 3i and Figure 4i). By adding a random constant between 0.1 and 0.5, the saturation and brightness of the images are modified. Before this, the images are converted from RGB to HSV color space. The resulting images are shown in Figure 3j and Figure 4j.

##### Blurring and Sharpening

For blurring, a Gaussian filter is applied to the image with a standard deviation between 2 and 4. Sharpening is performed by using a 3 × 3 filter kernel with randomly generated weights.

##### Histogram Equalization

When using the histogram equalization (see Figure 3j and Figure 4j) the intensity distribution of the image is transformed to an equal distribution. It is applied to improve the contrast of an image. The aim is to stretch the intensity values for the display of the image. This improves the visibility of details within the image which otherwise would be hard to be seen. In this paper, the histogram equalization implementation of openCV is used. Before applying it the image has to be converted to the YCbCr color space.

### 2.2. Hardware Setup

In this section, the hardware setup for recording the data, performing the sensor fusion and training the neural networks is presented.

#### 2.2.1. Thermal Camera

We used the thermal camera Optris PI 640 (by the company Optris, Berlin, Germany) with a field of view of 90${}^{\circ }$× 64${}^{\circ }$. The camera has an optical resolution of 640 × 480 pixels. It works within the wavelength spectrum between 7.5 μm and 13 μm. We use the measurement range of −20 ${}^{\circ }C$ to 100 ${}^{\circ }C$. The thermal sensitivity is 75 mK. The ROS package *optris_drivers* is used as driver [70].

#### 2.2.2. RGB Camera

The RGB camera used is the JAI GO-2400C-USB Camera with a Fish-Eye lens FUJINON FE185C086HA-1 by Sony (Tokyo, Japan). It has 7.5 μm square pixels and a field of view of 185${}^{\circ }$× 185${}^{\circ }$. The driver used was *ros_cvb_camera_driver* [71]. The resulting image has a size of 1146 × 716 pixels.

#### 2.2.3. 3D-Time-of-Flight Camera

We use a 3D time-of-flight camera called CamBoard pico flexx by the company pmd (Siegen, Germany). The camera has a field of view of 62${}^{\circ }$× 45${}^{\circ }$ and a resolution of 224 × 171 (38k) pixels. The measurement range is 0.1–4 m. A frame rate of up to 45 frames per second (fps) (3D frames) is delivered. We set a frame rate of 35 fps in our case. The camera can be connected via USB to an Ubuntu 18.04 computer with Robot Operating System (ROS) Melodic installed. The Royale SDK provided by the manufacturer as well as the ROS package *pico_flexx_driver* are the drivers used [72].

#### 2.2.4. Computer with GPU

An OMEN HP computer with Ubuntu 18.04.6 bionic operating system and a GeForce RTX 1080 Ti GPU from NVIDIA (NVIDIA, Santa Clara, CA, USA) (11 GB Memory) is used. We used the NVIDIA drivers 465.19.01, CUDA 11.3, cuDNN. We had the Melodic ROS version installed for recording the camera data.

### 2.3. Software Algorithms

All software algorithms for the sensor fusion as well as the face detection of our approach will be described in the following.

#### 2.3.1. Calibration

For the intrinsic calibration the ROS-package *camera_calibration* was used [73]. There is a special calibration program for the fish eye optics of the RGB camera [74].

##### Thermal Camera

The calibration target we used was made up of a 2 mm thick aluminum plate and a 1 mm thick high density fibreboard from which 6 × 20 circles with a diameter of 1 cm were cut out with a laser cutter. The distance between the circle centers is 13.96 mm. The calibration target was placed in the image with a horizontal orientation such that the long edge lies horizontally and the short edge vertically within the image. In total, 144 images were read. For the calibration the ROS package *camera_calibration* was used.

Concomitantly, *rqt_reconfigure* needs to be started so that the temperature threshold can be set. In this case, the temperatures are coded in black and white. Depending on the temperature of the calibration target the suitable threshold varies in order to make all circles of the calibration target visible.

**Figure 5:**
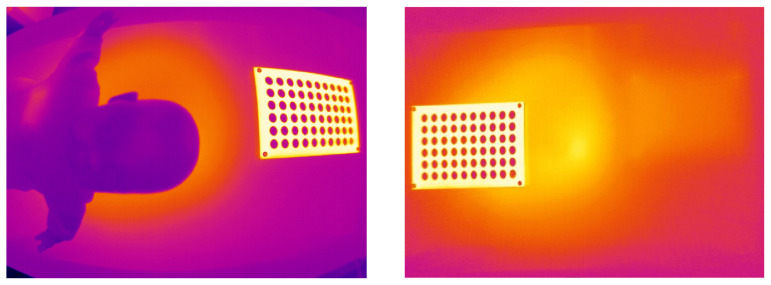
Intrinsic calibration of the thermal camera with 144 images. Rectified image (**right**) and non-rectified image (**left**). The cushion effect is sufficiently removed.

This resulted in a very good rectification of the image (see Figure 5). The cushion effects at the edges of the image are completely removed and the calibration target is visualized as rectangle within the thermal camera image. This was tested at different areas within the image. Using a baby doll, the occurrence of proximity-related distortions was excluded.

##### RGB Camera

**Figure 6:**
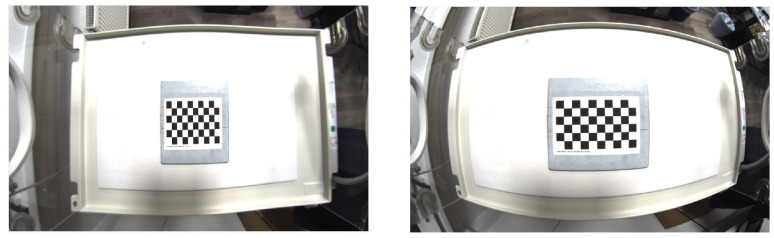
Intrinsic calibration with the cropped RGB camera image using 196 images (**left**). The calibration target is a 5 × 9 chess board with squares of size 14 mm. Non-rectified image (**right**).

First the RGB image was cropped to a size of 1146 × 716 pixels. The calibration was then performed with a 5 × 9 chess board with squares of size 14 mm. The result can be seen in Figure 6.

**Figure 7:**
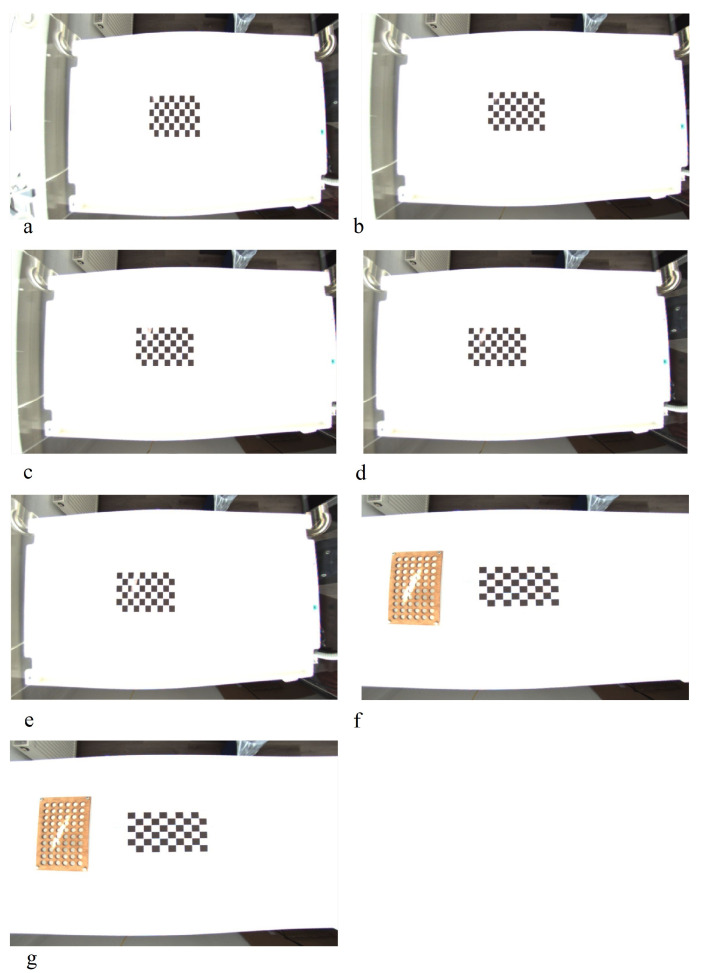
Empirical adaption of the intrinsic calibration of the RGB camera. (**a**) original image; (**b**) change the first value of the projection matrix from 337.7501220703125 to 400; (**c**) change the first value of the projection matrix from 337.7501220703125 to 410; (**d**) change the second value of the projection matrix from 567.37183155695675 to 500; (**e**) change the third value of the projection matrix from 456.1886901855469 to 470; (**f**) projection matrix with first value 560, second value 500 and third value 470; (**g**) change distortion matrix to [−0.2282522216659725, 0.03337792115699235, 0.0010099526756546235, −0.0010636239771667497, 0] and previous projection matrix.

In the last step, the values of the calibration were manually adapted such that the cushion effect decreases. It was empirically determined how the image changes when changing the projection as well as the distortion matrix. The step by step adaption can be seen in Figure 7.

#### 2.3.2. Thermal-RGB-Fusion

##### Circle Detection

**Figure 8:**
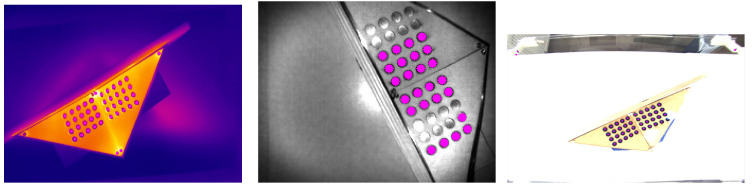
Blob detection within the thermal (**left**), ToF (**middle**) and RGB camera image (**right**). For the RGB image the contrast had to be increased by placing a blue paper sheet underneath.

For the detection of the circle centers, blob detection was used. Blob detection delivers so called keypoints which hold the center coordinates of the blob [75,76]. The results can be seen in Figure 8.

##### RGB-ToF and Thermal-ToF Fusion

The pixel indices of the ToF camera image and the point cloud correspond to each other due to the underlying internal fusion. By ordering the point cloud the corresponding 3D point of each pixel can be determined. In order to calculate the transformation, the 3D ToF points and the 2D thermal and RGB points are inserted into the function *SolvePnP*. The function calculates the orientation and position of the object from the given object points (3D ToF points) and image projections (2D thermal and RGB points), as well as the camera matrix and the distortion coefficients [77]. Internally, the Direct Linear Transform (DLT) algorithm is applied which calculates the matrix projecting the 3D ToF points into the 2D image plane.

In order to receive the rotation matrix from rvec the openCV function *Rodrigues* can be used [78]. It calculates the corresponding matrix from the vector. For applying the calculated transformation on the ToF data and to project the points into each image the function *projectPoints* is applied [79].

**Figure 9:**
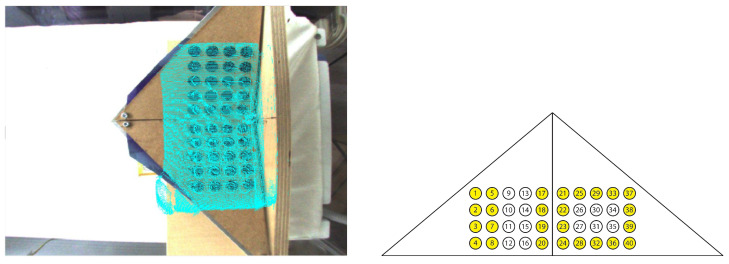
Extrinsic calibration of the RGB with the ToF camera; 26 point correspondences were used (**right**). The turquoise points are the projected ToF points (**left**).

**Figure 10:**
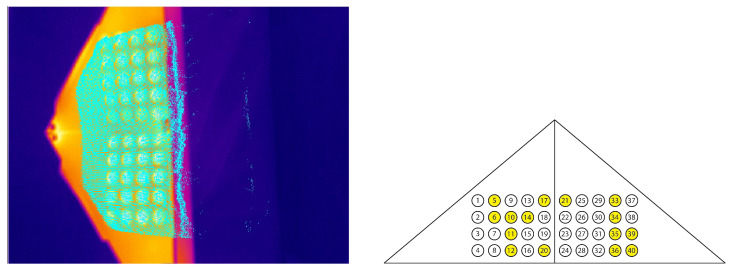
Extrinsic calibration of the thermal with the ToF camera; 15 point correspondences were used (**right**). The turquoise points are the projected ToF points (**left**).

In the following, the point correspondences which were used to calculate the RGB-ToF fusion (26 correspondences, see Figure 9) and the thermal-ToF fusion (15 correspondences, see Figure 10) are marked and the projected points into the images are shown. The most suitable combination of point correspondences was found empirically.

In order to achieve a very high fusion precision significant points from the video of the neonate can be included as point correspondences. In our case, individual adaptions had to be made for each subject.

**Figure 11:**
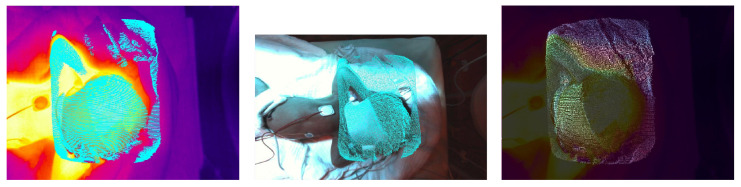
Thermal-ToF-fusion (**left**), RGB-ToF fusion (**middle**) and thermal-RGB-fusion camera image (**right**).

Once the extrinsic transformations are known, the thermal-RGB-fusion can be calculated. For each ToF point within the point cloud the corresponding RGB and thermal pixels are determined (see Figure 11). Then, the color information of the RGB pixel is inserted at the corresponding point within the thermal image, delivering the final fusion image. The size of the fusion image is the same as the thermal camera image.

#### 2.3.3. Neural Networks RetinaNet and YOLOv3

##### RetinaNet

In this paper, the RetinaNet implementation of the Git repository of fizyr is used [80], supporting *keras* and *tensorflow* (v2.4. were employed). For functional GPU support, some software packages needed to be installed. This includes NVIDIA drivers (driver 465.19.01), *CUDA* 11.3, *cuDNN* and *OpenCV* 4.5.254.

##### YOLOv3

In this paper, the Git repository of AlexeyAB is used [81]. For a smooth use of YOLOv3, several software requirements need to be met (details see above).

### 2.4. Measurement Series

The datasets holding thermal, RGB and ToF data were recorded at the Department of Pediatrics and Adolescent Medicine, University of Erlangen-Nürnberg, Germany. The study was conducted in accordance with the Declaration of Helsinki, and approved by the Ethics Committee of the Friedrich-Alexander Universität Erlangen-Nürnberg (protocol code: 197_20B and date of approval: 28 April 2021, amendment protocol code: 20-197_1-B, approved on 22 December 2022).

#### 2.4.1. Subjects

The subjects included in this study (see Table 1) were neonates of a minimum gestational age of 34 + 0 (late pre-term) that were clinically stable from a cardio-respiratory perspective. All subjects required phototherapy due to jaundice. The recordings took place in parallel to phototherapy.

#### 2.4.2. Training, Validation and Test Datasets

Our training datasets contain 1400 labeled images plus 1400 augmented images per each modality from subject 01 to 04. Our validation dataset contains 300 images from each modality of subject 01 to 04 which were not included in the training dataset. Table 2 shows the number of instances per class per modality.

The validation dataset was used to calculate the levels of average precision during the training process. With the test dataset we evaluate the performance of our trained networks. As test dataset 65 random images of each image modality of subject 05 were used which have not been seen by the networks yet. It is important that the distribution of the test dataset is similar to the training dataset.

## 3. Results

In this section, the training results for each modality and network are presented. Each network was trained for 64 epochs and the epoch with the best precision was determined. First, we show the quantitative results, where we compare the average precision per class. In the second step we present the test results with our test dataset.

### 3.1. RGB Dataset

In this section, we describe the results for the RGB dataset.

#### 3.1.1. RetinaNet

**Figure 12:**
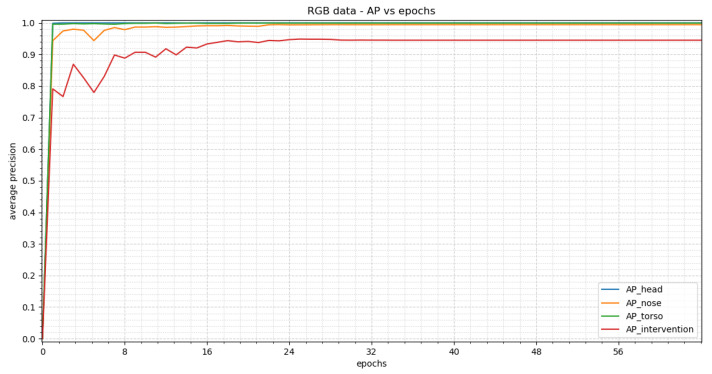
The training results for the RGB images achieved by RetinaNet can be seen in this figure. The average precision (AP) vs. epochs for the different classes are displayed. The trend shows a steep rise at the beginning which results in a stable plateau in epoch 25.

Figure 12 shows the average precision vs. epochs for the different classes within the RGB images. The best results are achieved in epoch 25. In this epoch, we obtain an average precision of 1.0 for the head, 0.9937 for the nose, 0.99 for the torso and 0.94 for the intervention.

**Figure 13:**
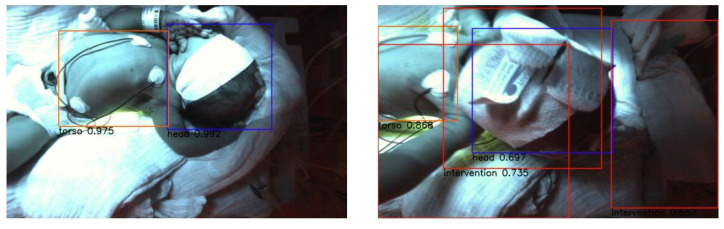
Visual evaluation of two example RGB images with RetinaNet. Good case (**left**) with confidence scores 0.992 for head and 0.975 for torso. Bad case (**right**) where the head is also detected as intervention with confidence scores 0.697 for head, 0.868 for torso and 0.735 for intervention. The false detection probably occurs due to the shorter distance between the face and the camera. This case is not sufficiently represented within the training dataset.

We used the weights of epoch 25 to evaluate our test dataset. We picked two representative images to visualize the detections (see Figure 13). The corresponding confidence scores are shown within the image. We achieved APs of 0.9255, 0.0, 0.7841 and 0.5884 for the classes head, nose, torso and intervention, respectively, over the whole test dataset.

#### 3.1.2. YOLOv3

**Figure 14:**
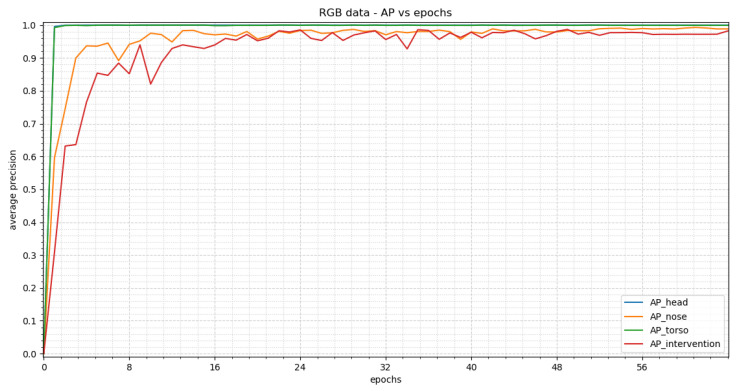
The training results for the RGB images reached by YOLOv3 can be seen in this figure. The average precision (AP) vs. epochs for the different classes are displayed. The trend shows a steep rise at the beginning and an oscillating behavior for class “nose” and “intervention”. The best precision values for all classes were achieved in epoch 64.

Figure 14 shows the average precision vs. epochs for the different classes within the RGB images for YOLOv3. The best precision is reached in epoch 64. In this epoch we receive APs of 1.0, 0.9885, 0.9991 and 0.9821 for head, nose, torso and intervention, respectively.

**Figure 15:**
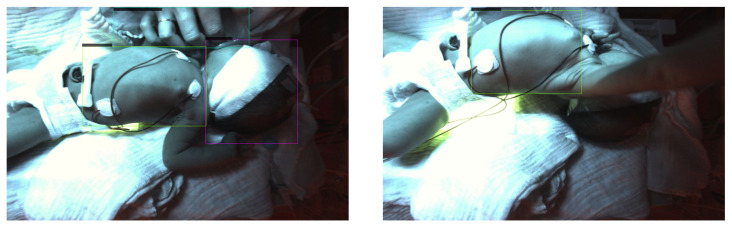
Visual evaluation of two example RGB images with YOLOv3. Good case (**left**) with confindence scores of 0.95 for the head, 0.99 for torso and 0.58 for the intervention. Bad case (**right**) where the head is not detected caused by the occlusion by the arm. Another factor is the twisted pose of the neonate’s body. This leads to a confidence score of 0.30 for the torso.

The weights of epoch 64 for evaluating our test dataset were used. Figure 15 shows two representative images to visualize the detections. The corresponding confidence scores can be found within the image description. We reach APs of 0.8563, 0.0, 0.8301 and 0.536 for the classes head, nose, torso and intervention, respectively, over the complete test dataset.

### 3.2. Thermal Dataset

In this section, the results for the thermal dataset are presented.

#### 3.2.1. RetinaNet

**Figure 16:**
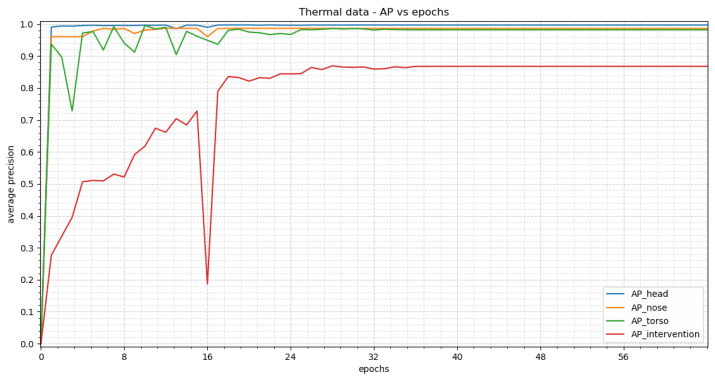
The resulting average precision values for the thermal images trained with RetinaNet are shown within the figure. First, a steep incline can be seen with the exception of the class “intervention” which shows a slower rise. In epoch 16, a collapse can be noted. By epoch 28 the precision values result in stable plateaus.

In Figure 16 the average precision vs. epochs for the different classes within the thermal images can be seen. Epoch 28 delivers the best results. We achieve an average precision of 0.9969, 0.9864, 0.9862 and 0.8695 for head, nose, torso and intervention, respectively.

**Figure 17:**
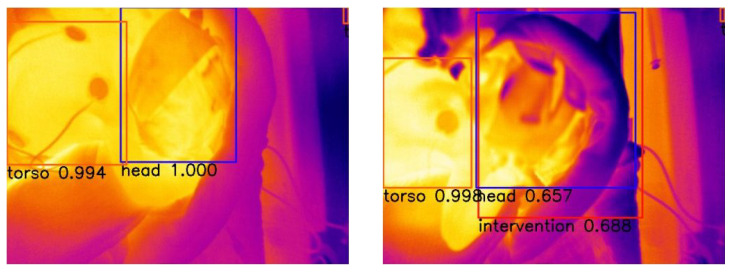
Visual evaluation of thermal images with RetinaNet. Good case (**left**) with confidence scores 1.0 for head and 0.994 for the torso. Bad case (**right**) where the head is also detected as intervention. The false detection occurs as the cloth surrounding the neonate’s face has a similar temperature as an adult’s, and is therefore detected as intervention. This leads to confidence scores of 0.657 for the head, 0.998 for the torso and 0.688 for the intervention.

To evaluate our test dataset the weights of epoch 28 were used. Figure 17 shows two representative images to visualize the detections. The corresponding confidence scores can be found within the image. For the evaluation of our whole dataset we receive APs of 0.9227, 0.0, 0.7816 and 0.4856 for the classes head, nose, torso and intervention, respectively.

#### 3.2.2. YOLOv3

**Figure 18:**
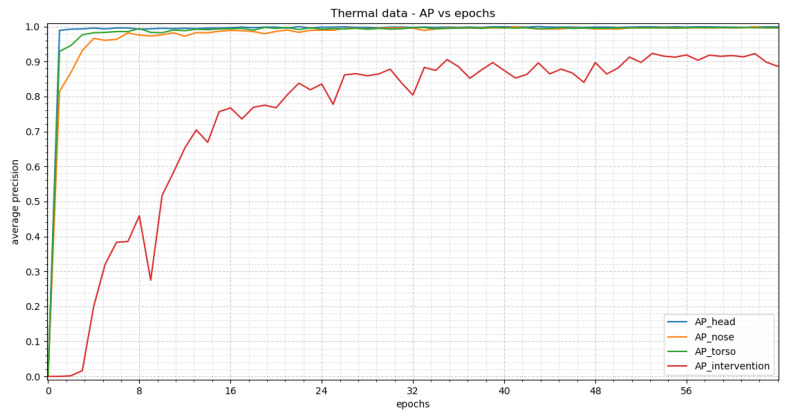
This figure shows the results for the training of YOLOv3 with thermal images. A steep increase can be seen at the beginnig for all classes other than “intervention”. “Intervention” shows an oscillating behavior.

Figure 18 displays the average precision vs. epochs for the different classes within the thermal images for YOLOv3. The best results occur in epoch 61. There APs of 0.9983 for the head, 0.9993 for the nose, 0.9963 for the torso and 0.9225 for the intervention are achieved.

**Figure 19:**
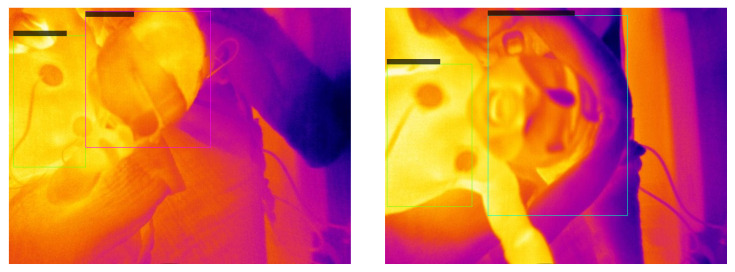
Visual evaluation of thermal images with YOLOv3. Good case (**left**) with confidence scores of 1.0 for the head and 0.95 for the torso. Bad case (**right**) where the head is detected as intervention with confidence scores of 0.99 for the torso and 0.25 for the intervention. The reasons are the same as mentioned for RetinaNet.

We used the weights of epoch 61 to evaluate our test dataset. Figure 19 displays two representative images to visualize the detections. The corresponding confidence scores are shown within the image description. We receive APs of 0.8924, 0.0, 0.8611 and 0.5706 for the classes head, nose, torso and intervention, respectively, over the complete test dataset.

### 3.3. Fusion Dataset

In this section, we describe the results for the fusion dataset.

#### 3.3.1. RetinaNet

**Figure 20:**
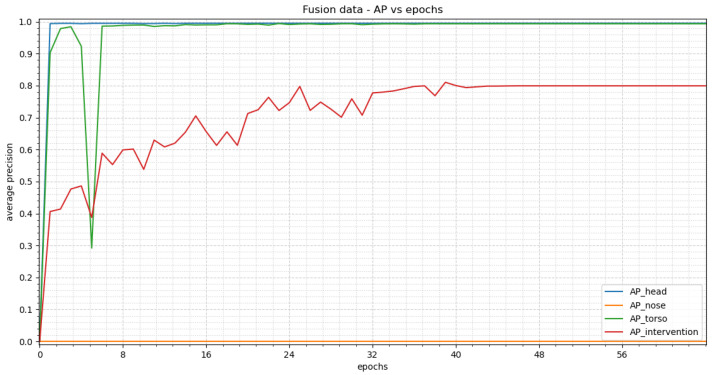
This figure displays the training results of RetinaNet with fusion images. The trend of “head” and “torso” has a steep incline at the beginning, resulting in a stable plateau in epoch 7. Nonetheless, there is a strong decline in epoch 5 for “torso”. “Intervention” shows a slower rise, resulting in a plateau in epoch 38. Due to the insufficient amount of instances of “nose”, the results cannot be taken into account. A further discussion of this issue can be found in Section 4.

Figure 20 shows the average precision vs. epochs for the different classes within the fusion images. In this case, the best epoch is number 38. We receive average precision values of 0.9949 for the head, 0.0 for the nose, 0.9934 for the torso and 0.7683 for the intervention.

**Figure 21:**
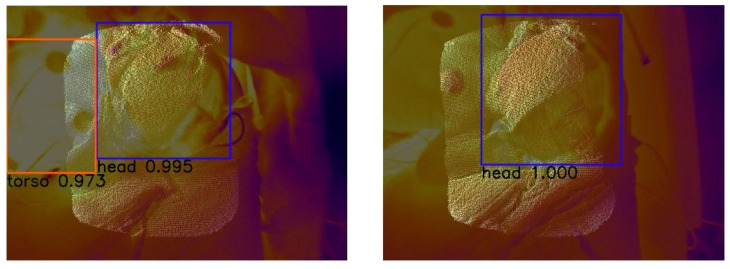
Visual evaluation of two fusion images with RetinaNet. Good case (**left**) with confidence scores of 0.995 for the head and 0.973 for the torso. Bad case (**right**) where the torso is not detected with a confidence score of 1.0 for the head. This is most likely caused by the small overlap of the torso and the ToF pointcloud due to a twisted pose of the neonate. Those images were not represented in our training dataset.

Figure 21 displays two representative images to visualize the detections. For the evaluation of our test dataset the weights of epoch 38 were used. The corresponding confidence scores can be seen within the image. We reached APs of 0.9958, 0.0, 0.6863 and 0.6574 for the classes head, nose, torso and intervention, respectively, over the whole test dataset.

#### 3.3.2. YOLOv3

**Figure 22:**
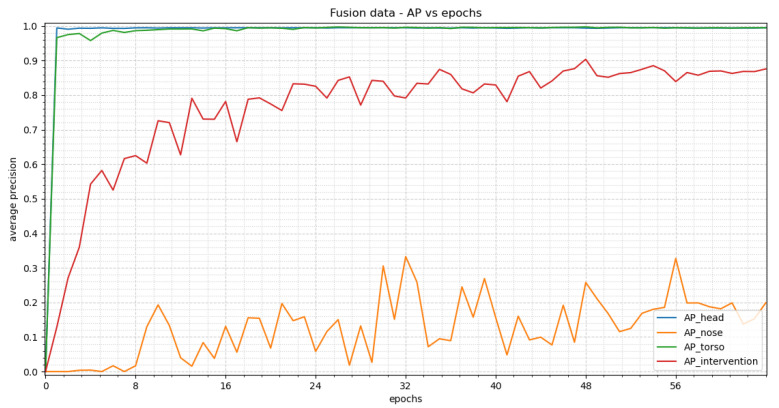
The results of YOLOv3 trained with fusion images can be seen in this figure. At the beginning a steep rise can be seen for “head” and “torso”, resulting in a stable plateau in epoch 24. “Nose” and “intervention” both show an oscillating behavior. “Intervention” has overall higher precision values than “nose” and its oscillation has a smaller amplitude. The precision values for “nose” cannot be taken into account due to an insufficient amount of class instances.

In Figure 22, the average precision vs. epochs for the different classes within the fusion images for YOLOv3 can be seen. Epoch 56 delivers the most precise findings. In this epoch, we obtain average precision values of 0.9949 for the head, 0.3274 for the nose, 0.9948 for the torso and 0.8390 for the intervention.

**Figure 23:**
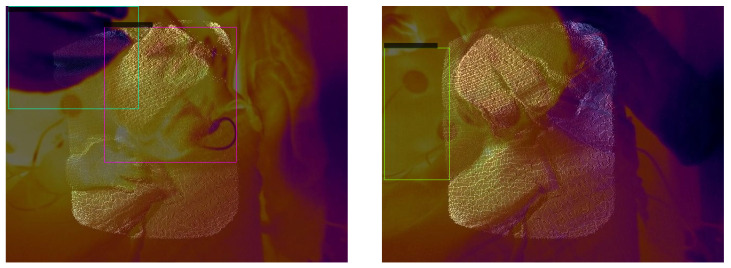
Visual evaluation of two sample fusion images with YOLOv3. Good case (**left**) with confidence scores of 0.95 for the head and 0.94 for the intervention. Bad case (**right**) where the head is not detected. The confidence score for the torso is 0.40.

Figure 23 shows two representative images to visualize the detections. Our test dataset was evaluated using the weights of epoch 56. The corresponding confidence scores can be found within the image description. We reach APs of 0.9455, 0.0, 0.7864 and 0.5199 for the classes head, nose, torso and intervention, respectively, over the complete test dataset.

## 4. Discussion

In this section, we discuss the results of the sensor fusion as well as the training results. Finally, we compare our results to the state of the art.

### 4.1. Comparison of Theoretical Approach for the Sensor Fusion

When we performed our sensor fusion approach we expected that the procedure only has to be undertaken once and that the extrinsic transformation can be applied to all subjects. This was not the case, most probably as small changes of the neonatal head and body position have a great impact due to the proximity of the cameras to the subject.

### 4.2. Discussion of Training Results

When discussing our training results we refer to the average precision values in Table 3.

#### 4.2.1. RetinaNet

When comparing the average precision with its trend of class “head” for all modalities almost identical results can be seen (smaller than 1%, see Table 4). The trend of the class “torso” within the RGB image is very similiar to the class “head” curve as can be seen in Figure 12. The same holds true for the fusion with the exception of one epoch where the precision drops significantly (see Figure 20). As this is only the case for one epoch this might be due to a wrong adaption of the weights at the beginning of the training. The thermal data shows an oscillating behavior until it reaches a stable value. This is due to the adjustment of the learning rate during the training process. The optimizer used adapts the step length such that it decreases the closer the optimum. A similar collapse can be seen for “intervention” within the thermal images (see Figure 16).

When looking at the trend of the “intervention” over all modalities we can see major differences in the precision compared to the “head” and “torso” (see Table 4). Furthermore we observed a relationship between the number of instances per class within the validation dataset and the reached precision i.e., 160 instances within RGB images leads to an AP of 0.94, 123 instances within thermal images gives an AP of 0.8695 and 80 instances within fusion images results in an AP of 0.7683. Therefore, we expect that an increase of instances of “intervention” within the thermal and fusion images will lead to higher precision. The same behavior can be seen for the “nose” within the fusion images where only right instances of the nose were included. Therefore, the precision values for the “nose” should not be taken into account. For the thermal and RGB images 300 “nose” instances within the dataset are enough to reach a similar precision as for the “head” and the “torso”.

As the fusion images hold more information than the RGB and thermal images by themselves the training takes more time (around 10 more epochs) to achieve the same precision.

The results generated by the test dataset show an overall decrease in the average precision. The reason for this lies in the complexity of some of the test images which were not present in the training dataset. This can especially be seen for the torso where often only the back or the side was visible due to the neonate lying in a twisted pose. This case was not covered by the training dataset. The training dataset also did not contain new forms of intervention such as the appearance of a nursing bottle. Additionally, the number of instances of the class “intervention” in the training dataset is low, leading to an overall worse performance. As before, the number of instances of the class “nose” is too low, resulting in an AP of 0.0 for all modalities. Clinically the most significant class is the head as the temperature and heart rate detection will depend on it. There were only small differences in the average precision values (improvement of 0.9% for the fusion, decrease of 4.42% for the thermal images and 7.45% for the RGB images). This shows that the fusion performs best. Due to the small number of test subjects included in the study a slight overfitting tendency was observed. We expect the average precision values to reach values comparable with the validation dataset once more subjects are included in the training process.

#### 4.2.2. YOLOv3

For YOLOv3 the classes “head” and “torso” deliver identical precision values for all modalities (difference less than 1%, see Table 5). As with the RetinaNet analysis, a direct relationship was observed in the validation images between the number of instances for the “intervention” class and the achieved average precision values. This means 160 instances within RGB images leads to an AP of 0.9821, 123 instances within thermal images gives an AP of 0.9225 and 80 instances within fusion images results in an AP of 0.8390. As before there are not enough instances of class “nose” within the fusion dataset to achieve precise detection. Therefore, these precision values should not be taken into account. The trends for “intervention” and “nose” show a fluctuating behavior. This can be explained by the usage of the Stochastic Gradient Descent (SGD) optimizer and its learning rate. (The optimizer used adapts the step length such that it decreases the closer the optimum).

Similar to RetinaNet an overall decrease in the average precision with the test dataset can be observed for YOLOv3. As the same test dataset was used the same issues occur as described before. These arise due to the complexity of the test images and new forms of intervention which were not taken into account before (e.g., nursing bottle). Another problem is the low number of instances of the classes “nose” and “intervention”. For the class “head” a decrease of 14.4% for the RGB, a decrease of 10.6% for the thermal images and 4.94% for the fusion images in the average precision values were observed. The data exhibit an overfitting trend due to the small number of test subjects. As mentioned before, we expect our average precision values to reach values similar to those of the validation dataset when using more test subjects for training.

#### 4.2.3. Comparison between RetinaNet and YOLOv3

When comparing results from RetinaNet and YOLOv3 for the validation fusion data, almost identical average precision values are reached for the classes “head” (difference of 0%) and “torso” (difference of 0.14%). For class “intervention” YOLOv3 has an increase in precision of around 7% compared to RetinaNet (see Table 6). Even though YOLOv3 reaches an average precision of 0.3274 for the “nose”, this result should be discounted, as it is caused by the small number of instances within the class. RetinaNet needs fewer epochs to achieve the highest precision values than YOLOv3. When taking into account the oscillating behavior of YOLOv3, as well as distinguishing between the different classes, we see that a similar number of epochs lead to stable precision values for RetinaNet when compared with YOLOv3.

As previously stated, both networks exhibit less favourable performances when used on the test dataset compared with the validation dataset due to the aforementioned reasons. Depending on the class one of the two networks performs slightly better (see Section 3).

Redmond et al. [30] and Hennemann [69] mention that the computing time per image is around three times greater for RetinaNet than YOLOv3. A similar difference was observed in our study. We reached approx. 58 ms and approx. 14 ms for RetinaNet and YOLOv3 on our hardware setup.

#### 4.2.4. Summary

In summary, the fusion network is able to deliver the same precision values as the RGB and thermal networks. This is the case if there is an equal number of images and instances per class for each modality and class. If the extrinsic transformation between the thermal and RGB camera is given, the position of the detected region can automatically be calculated for the RGB and the thermal image. A necessary condition is that the region of interest (ROI) lies within the ToF pointcloud. This results in the great advantage that only one network needs to be trained and, therefore, no labeling of the RGB and thermal data is needed. This simplifies the generation of datasets with respect to time and cost (we save 66%).

As shown by Hennemann, better results over all classes can be achieved by training with a greater number of test subjects [69].

When comparing the average computing time of the two networks, YOLOv3 performs slightly better in our setup. When taking the average precision values into account, we observed only marginal differences in average precision values between classes. We therefore believe that both networks are adequate for neonatal face detection.

### 4.3. Comparison with State of the Art

First we compare our sensor fusion approach to the methods mentioned in Section 1.1. We describe how our results using a neural networks training approach compare with other neonatal face detection networks.

#### 4.3.1. Sensor Fusion

To our knowledge, our sensor fusion approach is the first which is explicitly designed for the short distances commonly encountered in the setting of incubator nursing of premature neonates on the NICU. At the time of completion of this work, no literature regarding the use of ToF camera as third sensor for indirect fusion was found.

#### 4.3.2. Neural Network for Face Detection

In this section, we compare our results with the best performing findings in the literature for each class mentioned in Section 1.1. Green et al. reach an average precision of 0.982 for the detection of the head [35]. Our fusion approach achieves a precision of 0.9949 (RetinaNet) and 0.9949 (YOLOv3). For the test dataset we reach 0.9958 and 0.9455 for RetinaNet and YOLOv3, respectively. This shows that we achieve similarly high precision values for instances of the “head” class within data the neural network has not seen before. For “nose” and “intervention” there are no precision values available within the literature. Beppu et al. receive an AP of 0.985 for the torso [49]. We observe precision values of 0.9934 (RetinaNet) and 0.9948 (YOLOv3) with the fusion network. When considering our test dataset we achieve APs of 0.6863 (RetinaNet) and 0.7864 (YOLOv3). As mentioned before, we expect these values to increase to similar precision values when training with more test subjects. As we can directly calculate the detections for the RGB and thermal images, we will also receive such high precision. So far, we are the first to report the use of thermal-RGB-fused images of neonates to train a neural network.

We hope our results will facilitate the development of non-contact neonatal monitoring techniques to improve the standard of care for this vulnerable group of patients.

## 5. Conclusions

In this contribution, we investigated the use of thermal-RGB-fusion data for a robust neonatal face detection method based on neural networks. We showed that it is possible to achieve a precise sensor fusion for short distances using a ToF camera as third sensor. We trained two different neural networks (RetinaNet and YOLOv3) with thermal, RGB and fusion images and evaluated the precision values achieved. Our fusion networks deliver the same level of precision as the RGB and thermal networks as long as an equal number of instances per class and modality are available (at least 300) within the dataset. Compared to the state of the art we achieve comparable average precision values for the head. Based on the known extrinsic calibration between our cameras we can easily calculate the detection within the thermal and RGB images from the fusion network. Therefore, this increases data efficiency (training of a single network, reduction of data labelling by 66%) and economizes the process. Both RetinaNet and YOLOv3 could be used for detection of the facial region in fused images of neonates in the clinical setting. For the improvement of the generalization of the neural network we intend to train with more subjects in the future.

## Figures and Tables

**Table 1:** List of subjects. Term neonates were > 37 + 0 weeks of gestation.

Subject	Gestational Age	Age during Study	Sex	Weight
01	34 + 0	2 days	male	1745 g
02	term	5 days	female	3650 g
03	term	4 days	female	2330 g
04	term	13 days	male	3300 g
05	term	2 days	female	2750 g

**Table 2:** Number of instances per class per modality within the validation dataset.

Modality	Head	Nose	Torso	Intervention
**RGB**	1193	575	1129	160
**Thermal**	1199	305	1183	123
**Fusion**	1190	8	1055	80

**Table 3:** Average precision values for different classes using different modalities and networks (validation dataset).

	Modality	Epoch	Average Precision			
			Head	Nose	Torso	Intervention
**RetinaNet**	**RGB**	25	1.0	0.9937	0.99	0.94
	**Thermal**	28	0.9969	0.9864	0.9862	0.8695
	**Fusion**	38	0.9949	0.0 *	0.9934	0.7683
**YOLOv3**	**RGB**	64	1.0	0.9885	0.9991	0.9821
	**Thermal**	61	0.9983	0.9993	0.9963	0.9225
	**Fusion**	56	0.9949	0.3274 *	0.9948	0.8390

* The precision values for “nose” cannot be taken into account due to an insufficient number of class instances within the fusion dataset.

**Table 4:** Difference between fusion and RGB/thermal in average precision values per class for RetinaNet (validation dataset).

	Modality	Epoch	Average Precision			
			Head	Nose	Torso	Intervention
**RetinaNet**	**Fusion—RGB**	13	−0.0051	−0.9937 *	0.0034	−0.1717
	**Fusion—Thermal**	10	−0.002	−0.9864 *	0.0072	−0.1012

* The precision values for “nose” cannot be taken into account due to an insufficient number of class instances within the fusion dataset.

**Table 5:** Difference between fusion and RGB/thermal in average precision levels per class for YOLOv3 (validation dataset).

	Modality	Epoch	Average Precision			
			Head	Nose	Torso	Intervention
**YOLOv3**	**Fusion—RGB**	−8	−0.0051	−0.6611 *	−0.0043	−0.1431
	**Fusion—Thermal**	−5	−0.0034	−0.6719 *	−0.0015	−0.0835

* The precision values for “nose” cannot be taken into account due to an insufficient number of class instances.

**Table 6:** Comparison of average precision values between classes and modalities RetinaNet—YOLOv3 (validation dataset).

	Modality	Epoch	Average Precision			
			Head	Nose	Torso	Intervention
**RetinaNet—YOLOv3**	**RGB**	−39	0.0	0.0052	−0.0091	−0.0421
	**Thermal**	−33	−0.0014	−0.0066	−0.0101	−0.053
	**Fusion**	−18	0.0	−0.3274 *	−0.0014	−0.0707

* The precision values for “nose” cannot be taken into account due to an insufficient number of class instances.

## Abbreviations

The following abbreviations are used in this manuscript:

AP	average precision
BPM	breaths per minute
CNN	Convolutional Neural Network
DLT	Direct Linear Transform
ECG	Electrocardiogram
FFT	Fast Fourier Transform
FoV	field of view
FPN	Feature Pyramid Network
fps	frames per second
GAN	Generative Adversial Network
GPU	graphical processing unit
IoU	Intersection over Union
IR	infrared
LIDAR	light detection and ranging
LSTM	Long Short-Term Memory
LWIR	long wave infrared
NCC	normalized cross correlation
NICU	Neonatal Intensive Care Unit
NIR	near infrared
PCA	Principal Component Analysis
PCL	Point Cloud Library
R-CNN	Region-Based Convolutional Neural Networks
ResNet	Residual Network
RGB	Red Green Blue
RGB-D	Red, Green, Blue -Depth
RMSE	root mean square error
ROI	region of interest
ROS	Robot Operating System
SGD	Stochastic Gradient Descent
SNR	Signal-to-noise ratio
T-ICP	thermal-guided iterative closest point
ToF	time-of-flight
UV	ultraviolet
YOLO	you only look once
